# Enhancing silicide formation in Ni/Si(111) by Ag-Si particles at the interface

**DOI:** 10.1038/s41598-019-45104-3

**Published:** 2019-06-20

**Authors:** Cheng-Hsun-Tony Chang, Pei-Cheng Jiang, Yu-Ting Chow, Hsi-Lien Hsiao, Wei-Bin Su, Jyh-Shen Tsay

**Affiliations:** 1 Department of Physics, National Taiwan Normal University, Taipei, 116 Taiwan; 2 Institute of Physics, Academia Sinica, Taipei, 11529 Taiwan; 3 Department of Electronic Engineering, Minghsin University of Science and Technology, Hsinchu, 30401 Taiwan; 4 Department of Physics, Tunghai University, Taichung, 407 Taiwan

**Keywords:** Magnetic properties and materials, Nanoparticles, Surfaces, interfaces and thin films

## Abstract

Compound formation at a metal/semiconductor interface plays crucial roles in the properties of many material systems. Applications of Ni silicides span numerous areas and have the potential to be used as new functionalities. However, the magnetic properties of ultrathin Ni layers on silicon surfaces and related chemical compositions at the interface are not fully understood and the influence of Ag additives on the reactivity of Ni/Si(111) remain unclear. We report herein on the fact that the dominant species produced at the interface is NiSi, which is produced by the spontaneous formation of strong bonds between Ni and Si atoms. Assuming that a Ni layer is formed over a NiSi layer with the total coverage as a constraint, we established a chemical shift-related concentration model that, in effect, represents a practical method for determining the amount of ultrathin Ni silicides that are produced at the buried interface. The formation of Ag-Si particles provide a viable strategy for enhancing silicide formation via a specific interaction transfer mechanism, even at room temperature. The mechanism is related to differences in the enthalpies of formation ΔH_Ag-Si_, ΔH_Ni-Ag_, and ΔH_Ni-Si_, for these phases and provides insights into strategies for producing ultrathin silicides at a buried interface.

## Introduction

Metal-semiconductor interfaces have attracted considerable interest because of their promising applications as semiconductor devices and possible use as new functionalities in semiconductor technology^1–6^. Owing to the high chemical reactivity of semiconductor surfaces, the formation of compounds at metal/semiconductor interfaces plays crucial roles in determining the properties of material systems and open a wide range of applications^5–17^. For example, cobalt di-silicide shows intrinsically low noise properties which could be utilized for developing superconducting circuits and qubits in the future quantum computing^8^. Metal silicides have been shown to have the ability of catalyze various reactions, especially hydrogenation, due to their specific crystal and electronic structures^10^. For higher conversion efficiencies of the optical absorption spectrum, *β*-FeSi_2_-containing SiO_2_ composites provide desirable selective absorbing behavior^9^. Moreover, the formation of compounds at metal/semiconductor interfaces are induced by impurities^16–19^. At elevated temperatures, the interaction transferring for Si atoms through a $(\sqrt{3}\times \sqrt{3})R{30}^{o}$**-**Ag layer causes a gradual interaction between Si and Co and the resulting smooth surface is advantageous in terms of stabilizing the easy axis of magnetization^16^. The distribution of substitution alloying elements in the γ-Nb_5_Si_3_, which serves as a reinforcing phase of the composite based on Nb–Si^18^. By implanting nitrogen under the critical conditions, the growth of metal-rich silicide phases is circumvented, as the Ni film converts to the amorphous NiSi phase during annealing^19^. Because of the interest in developing novel spintronic devices, explorations directed toward attempting to combine the charge and spin degrees of freedoms have increased dramatically^12–28^. Based on a successful combination of a solution process and the efficient control of the electric potential for magnetism, a novel concept of electric-potential-tuned magnetic recording has been developed, resulting in the development of stable recording media with a high degree of writing ability^20^. The formation of CoSi_2_ compounds at the interface on a Si(111) surface occurs and the easy axis of magnetization of Co/Si films is canted out-of-plane due to interfacial effects^14^. By tuning the contact areas of Ag and Ni at the Ag/Ni interface, it is possible to change the coercive force of the films by modifying the magnetic anisotropy energy^13^. By controlling interface band alignment, the functionality of the EuO/Si spin contact shows a conduction band offset of 1.0 eV, a value that is competitive with charge electronics^24^.

Nickel is widely used as catalyst, including for fabricating various nanostructures, and in water splitting, hydrogen production, and sulfurization processes^29–32^. Nickel is also widely used in steel alloys and electroplating^33–38^. As a magnetic material, nickel has an advantage of a relatively low Curie temperature (627 K), which permits it to be used in studies of critical phenomenon near room temperature^39–41^. The relatively small coercive force of nickel allows Ni-based thin films to be studied under relatively low external fields^42,43^. Silicon is the most important substrate that is used in fabricating microelectronic components. Because of the low resistivity and low fabrication temperature, Ni/Si systems are widely used in the electronics industry as ohmic contacts and interconnects^44,45^. Applications of Ni silicides span numerous areas, including their use in silicon complementary metal-oxide semiconductors^5,12^, field effect transistors^11^, batteries^46,47^ and floating gate memory^22^. Ni_2_Si, NiSi and NiSi_2_ all appear to be present at Ni/Si interfaces and the composition is dependent on the temperature of the sample and the preparation procedure^6,48,49^. There are relatively few reports dealing with the magnetic properties of ultrathin Ni layers on silicon surfaces and chemical compositions of these materials at the interface. In additional, the influence of Ag additives on the reactivity of Ni/Si(111) remain unclear. In this study, we report on an investigation of the morphologies, chemical states and related magnetic properties of Ni/Si(111). NiSi is the relatively dominant species found at the Ni/Si interface because of the strong bonding between Ni and Si atoms. Assuming that a Ni layer is formed over a NiSi layer with the total coverage as a constraint, we established a chemical shift-related concentration (CSRC) model that represents a practical method for determining the amount of ultrathin Ni silicides that are produced at the buried interface. For a submonolayer Ni deposited on a few monolayers of Ag/Si(111), the chemical state of Ni was found to be closer to that for Ni silicides rather than for Ni-Ag bonding. This is the first observation to confirm that Ag-Si particles serve as a catalyst to promote the chemical interaction of Ni and Si to form Ni silicides. The mechanism is related to the differences in the enthalpies of formation Δ*H*_Ag-Si_, Δ*H*_Ni-Ag_, and Δ*H*_Ni-Si_, and provide insights into strategies for producing ultrathin silicides at a buried interface.

## Results and Discussion

All experiments were conducted in an ultrahigh vacuum (UHV) chamber to avoid possible contamination from the residual gases. The Si(111) surface was cleaned by Ar^+^ ion bombardment and annealed at 1250 K. The sputtering-annealing cycles followed by slowly cooling to room temperature were continued until a well-ordered 7 × 7 structure could be obtained by low-energy electron diffraction (LEED) and scanning tunneling microscopy (STM) techniques^15,16^. The cleanliness of the specimen was checked by Auger electron spectroscopy (AES). Ni atoms were evaporated by passing an electronic current through a resistively heated Ni coil. The detailed information about the sample preparations are described in the method section as well as in our previous reports^13–17^.

**Figure 1:**
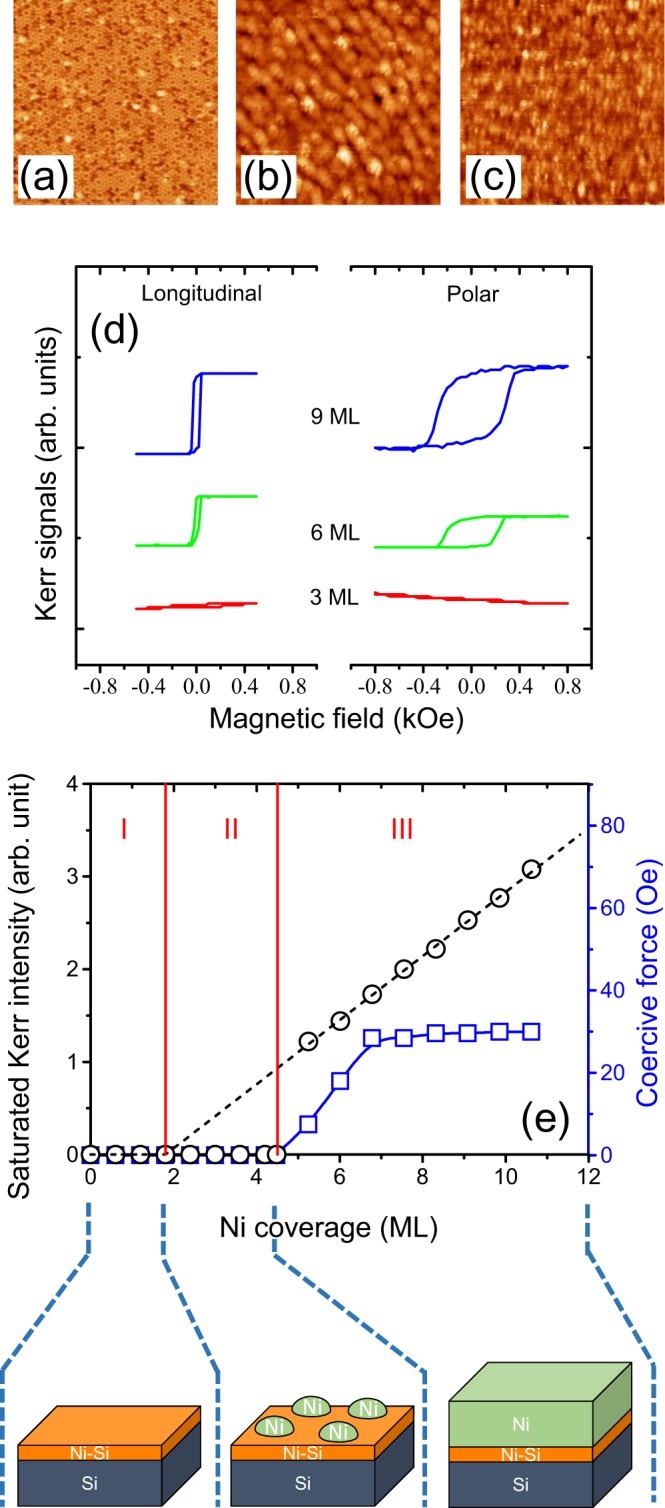
STM images of (**a**) 0.8, (**b**) 2.4, and (**c**) 5 ML Ni/Si(111). The images sizes are 50 × 50 nm^2^. (**d**) Kerr signals versus the magnetic field for 3, 6 and 9 ML Ni/Si(111) in both the longitudinal and polar configurations. (**e**) Saturated Kerr intensity and coercive force versus Ni coverage in the longitudinal configuration. The different regimes are characterized by different magnetic behaviors. They are composed of nonmagnetic silicides, clusters with lowered Curie temperature, and ferromagnetic films, as illustrated at the bottom of the plot.

During the deposition of Ni on Si(111)-7 × 7, the morphological evolution of Ni/Si interfaces was investigated using an STM technique. STM images for 0.8, 2.4, and 5 monolayer (ML) Ni/Si(111) are shown in Fig. 1a–c, respectively. For imaging the silicide related defects on the Si(111)-7 × 7 substrate, a negative bias potential (*V*_bias_) at −1.5 V was used. As shown in Fig. 1a for 0.8 ML Ni/Si(111), dark defects can be seen on the rhombic unit cell of the Si(111)-7 × 7 substrate. The dark defects are similar to that in Co/Si(111) for which the presence of Co silicides have been reported^14^. For 0.8 ML Ni/Si(111), the dark defects are related to the formation of Ni silicides. The dark defects have different sizes, which can be attributed to different chemical states of Ni silicides. For the case of 2.4 ML Ni/Si(111) in Fig. 1b, small clusters with oval shapes can be seen. This is due to the accumulation of Ni atoms to form clusters. For the case of the thicker Ni/Si(111) in Fig. 1c (5 ML), the surface morphology is rather smooth indicating the formation of a continuous film. These structural findings are important for the further analysis of magnetic properties of Ni/Si(111) ultrathin films.

Using the surface magneto-optic Kerr effect (SMOKE) technique at room temperature, Kerr signals versus the magnetic field in both the longitudinal and polar configurations are shown in Fig. 1d. At the initial stage of the Ni deposition (3 ML), no hysteresis is detectable in either of the configurations, but hysteresis clearly occurs at thicker Ni/Si(111) (6 ML). As the Ni coverage increases (9 ML), both the saturated Kerr intensities and coercive forces become larger. The coercive force in the case of the polar configuration is ten times larger than that in the longitudinal configuration. This result shows that the preferred easy axis of magnetization for Ni/Si(111) lies on the surface plane. Since the in-plane component dominates the magnetic anisotropy, in the following discussion, we focus on the magnetic properties only in the longitudinal configuration. Figure 1e shows the saturated Kerr intensity and coercive force versus Ni coverage in the longitudinal configuration. At the initial stage of Ni deposition, the Kerr intensity is zero for the first 4 ML while the Kerr intensity is nonzero at a thickness of around 5 ML. For Ni/Si(111) thicker than 5 ML, the Kerr intensity increases linearly with increasing Ni coverage. This shows that ferromagnetic films were formed with a Curie temperature well above room temperature where the SMOKE measurements were taken. In a straight-line extrapolation of the data for Ni thicker than 5 ML, the fitting line passes the transverse axis of Ni coverage at 1.8 ML. This indicates that the first-1.8-ML of Ni is nonmagnetic due to the formation of Ni silicides. This method for insure nonmagnetic layer is developing in our group and reported in many previous reports^14–17^. For Ni/Si(111) with a coverage between 1.8 and 5 ML, the zero value of the Kerr intensity can be attributed to the finite size effect resulting from the formation of Ni clusters^39,50^. The thermal agitation of the magnetization diminishes the exchange coupling between atomic magnetic moments in Ni clusters^13,15^. Therefore, Ni/Si(111) adopts a phase comprised of paramagnetic clusters. On the other hand, the coercive force increases rapidly from 7.5 to 30 Oe for 5 to 7 ML Ni and reaches a steady value of 30 Oe for thicknesses of up to 11 ML. This is due to the effects of reduced dimensionality, reflecting the evolution of the magnetic anisotropy energy for thin films^21,39,50,51^. The results of SMOKE measurements with three phases in different coverage regimes for ultrathin Ni/Si(111) films are shown at the bottom of Fig. 1e and include (I) nonmagnetic silicides, (II) clusters with lowered Curie temperatures, and (III) ferromagnetic films.

**Figure 2:**
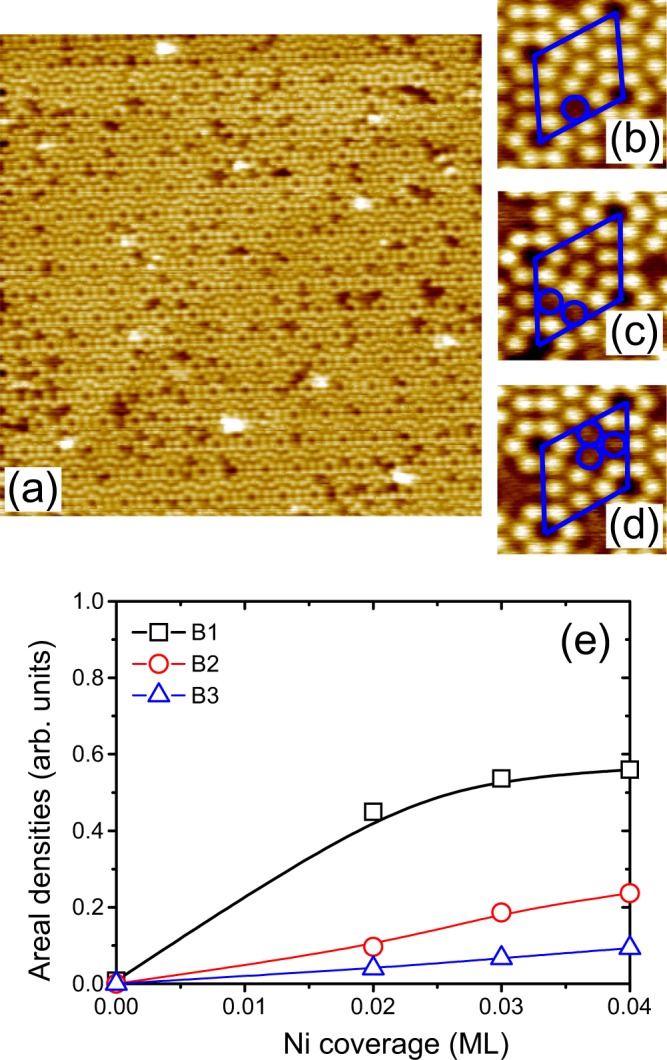
(**a**) STM images of 0.02 ML Ni/Si(111). Enhanced STM images showing (**b**) B1, (**c**) B2, and (**d**) B3. The image sizes are 50 × 50 nm^2^ for (**a**), and 1.0 × 1.0 nm^2^ for (**b**–**d**). (**e**) Statistics for the areal densities of B1, B2, and B3 calculated from STM images in 50 × 50 nm^2^ area for Ni adatoms on Si(111).

To obtain further information related to silicide formation for Ni adatoms on Si(111)-7 × 7, the morphological evolution of Ni/Si interfaces were explored using the STM technique. As an example, Fig. 2a shows an STM image for 0.02 ML Ni/Si(111) at a negative *V*_bias_. Different sized dark defects are clearly observed on the surface. This phenomenon is different from the Co silicide formation process for Co deposition on Si(111)-7 × 7, where only double-dark spots are detected, indicating that the dominant species in this case is CoSi_2_^14^. The appearance of different sized, dark defects for Ni deposited on Si(111)-7 × 7 suggests that different species of Ni silicides are produced. At the initial stage of Ni deposition on Si(111)-7 × 7, the number of defects increases with increasing Ni coverage. In order to carry out a quantitative analysis, the defects were categorized according to the number of dark spots in a unit cell, B1, B2, and B3, as shown in Fig. 2b–d, respectively. The areal densities for B1, B2, and B3 are defined as the ratios of B1, B2, and B3 unit cells per total unit cells, respectively. The statistics for the areal densities of B1, B2, and B3 are shown in Fig. 2e, calculated from STM images of 50 × 50 nm^2^ areas for small amounts of Ni atoms on Si(111). By increasing the coverage of Ni, the areal densities of B1, B2 and B3 increase while the slopes for these increases are different. After examining the STM images, single-dark spots (B1) occur randomly on the Si(111) surface. Ni adatoms have no particularly preferred site for their bonding on the rhombic unit cells of the Si(111)-7 × 7 substrate. The B1 spot is related to the formation of NiSi and can be attributed to the presence of strong bonding between Ni and Si atoms. In addition, the areal density of B1 is much larger than those for B2 and B3. This shows that NiSi is the relatively dominant species at the Ni/Si interface during the deposition of Ni. Because of the lower enthalpy of formation of NiSi compared to that for NiSi_2_^52^, it is reasonable to conclude that NiSi is the dominant species at the interface for the Ni coverage from submonolayer to continuous film. Spectroscopic evidence shows that the Ni silicides consist of only NiSi and NiSi_2_ for the case of Ni deposition on a Si substrate at room temperature^53^. We therefore conclude that the increases in the areal densities of B2 and B3 are due to an increased level of NiSi_2_, the accumulation of NiSi, and a mixture of NiSi and NiSi_2_.

**Figure 3:**
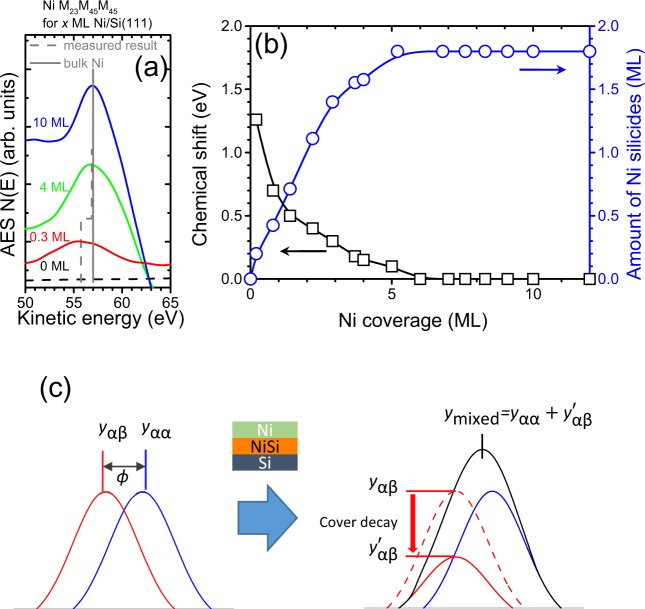
(**a**) Auger signals *N*(*E*) near the Ni M_23_M_45_M_45_ Auger transition versus kinetic energy for 0, 0.3, 4 and 10 ML Ni/Si(111). (**b**) Chemical shift (black squares) and amount of Ni silicides (blue circles) versus Ni coverage for Ni/Si(111) thinner than 12 ML. (**c**) Schematic plots for simulated Auger peaks by assuming α-α and α-β bonds with a layered structure.

Because of the finite inelastic mean free path (IMFP) of Auger electrons^54,55^, AES is sensitive to chemical interactions that occur at the Ni/Si interface. Figure 3a shows Auger signals *N*(*E*) near the Ni M_23_M_45_M_45_ Auger transition versus the kinetic energy (*E*) for Ni/Si(111) for different levels of Ni coverage; where *N*(*E*) corresponds to the counts of the AES measurements at kinetic energy *E*. For the case of 10 ML Ni/Si(111), it is sufficiently thick compared to the IMFP of Ni M_23_M_45_M_45_ Auger electrons (~0.47 nm)^55^ and therefore the peak position of the Auger signals *N*(*E*) located at 57.0 eV is close to that of bulk Ni (grey solid line). For the case of thinner Ni/Si(111), the Ni M_23_M_45_M_45_ Auger transition line shifts to a lower kinetic energy and the peak position of the Auger signals *N*(*E*) reaches 55.7 eV for 0.3 ML Ni/Si(111). Because Si is more electronegative than Ni^56^, the lowered kinetic energy of the Ni Auger transition line confirms the formation of Ni-Si compounds at the interface due to a change in the chemical environment of the Ni atoms from Ni-Ni to Ni-Si (grey dash line). We define the chemical shift of Ni M_23_M_45_M_45_ as the difference in the position of the peak for the Auger signals *N*(*E*) respective to that for pure Ni. Figure 3b (black squares) shows the chemical shift for Ni M_23_M_45_M_45_ versus Ni coverage for Ni/Si(111) thicknesses less than 12 ML. For 0.3 ML Ni/Si(111), the chemical shift is as large as 1.3 eV, indicating that Ni silicide is formed at the interface. The chemical shift decreases with increasing Ni coverage. In order to explore the amount of Ni silicides at the interface, we propose a CSRC model based on analyses of the Auger peak position, as discussed below.

Auger signals *N*(*E*) for an adsorbate in the elemental form are located at a certain value. When there is a chemical interaction between the adsorbate and substrate atoms, the change in chemical state is accompanied by a shift in the position of the Auger signals *N*(*E*). We attempted to correlate the chemical shift with the composition of the compounds that were formed using the proposed CSRC model, as illustrated as a schematic plot in Fig. 3c. We simulated the Auger signal *N*(*E*) as a Gaussian function. The kinetic energy of such compounds differ from one another and can be simulated by a shift in the position in the Gaussian function. For element α with an α-α bond, the Auger signal can be expressed as

1 $${y}_{\alpha \alpha }(E)={I}_{\alpha \alpha }\cdot {e}^{-{E}^{2}}$$

where *I*_αα_ is the intensity related to the amount of element α with an α-α bond. Chemical interactions between elements α and β may result in a different in the energy *ϕ* and the related Auger signal can be expressed as

2 $${y}_{\alpha \beta }(E)={I}_{\alpha \beta }\cdot {e}^{-{(E+\varphi )}^{2}}$$

where *I*_αβ_ is the intensity related to the amount of element α with an α-β bond. During the process of compound formation, both the elemental state and the compound state are present and the shift of the Auger transition line depends on the relative compositions of each. If compound formation occurs at the interface, we assume that the α-α and α-β regions are distributed in a stacking arrangement, as shown in the Fig. 3c. The Auger intensity of α-α layers follows a homogeneous attenuation model^54^ and can be expressed as

3 $${I}_{\alpha \alpha }={I}_{0}(1-{e}^{-{d}_{\alpha \alpha }/\lambda })$$

where *d*_*αα*_ is the layer thickness; *λ* is the escape depth of the Auger electrons characterizing their IMFP in the solid; *I*_0_ is the Auger sensitivity. We assume that the Auger sensitivity (*I*_0_) is the same for both the α-α layers and α-β layers. By considering an α-β compound layer covered by an α-α layer, an ${e}^{-{d}_{\alpha \alpha }/\lambda }$ term can be added to Equation 3. The intensity *I*_*αβ*_ is then expressed as

4 $${I}_{\alpha \beta }={I}_{0}(1-{e}^{-{d}_{\alpha \beta }/\lambda })\cdot {e}^{-{d}_{\alpha \alpha }/\lambda }$$

The resulting Auger signal related to the thickness *d*_*αα*_ for α-α layers and *d*_*αβ*_ for α-β layers can be represented as

5 $$\begin{array}{rcl}{y}_{S} & = & {y}_{\alpha \alpha }+\,{y}_{\alpha \beta }\\  & = & {I}_{0}(1-{e}^{-{d}_{\alpha \alpha }/\lambda }){e}^{-{E}^{2}}+{I}_{0}(1-{e}^{-{d}_{\alpha \beta }/\lambda })\cdot {e}^{-{d}_{\alpha \alpha }/\lambda }\,{e}^{-{(E+\varphi )}^{2}}\end{array}$$

As shown in Equation (5), the Auger peak position (*E*_*p*_) can be obtained from the maxima value of the mixed Auger signal (*y*_*s*_) within the critical thickness of α-α and α-β bonds.

6 $$\begin{array}{rcl}{\frac{d{y}_{S}}{dE}|}_{E={E}_{p}} & = & -2{I}_{0}\cdot [E\cdot (1-{e}^{-{d}_{\alpha \alpha }/\lambda }){e}^{-{E}^{2}}+(E+\varphi )\cdot (1-{e}^{-{d}_{\alpha \beta }/\lambda })\cdot {e}^{-{d}_{\alpha \alpha }/\lambda }\\  &  & \cdot \,{e}^{-{(E+\varphi )}^{2}}]{|}_{E={E}_{p}}\,=0\end{array}$$

The Auger peak position *E*_*p*_ follows the Equation

7 $$\frac{(1-{e}^{-{d}_{\alpha \alpha }/\lambda })}{(1-{e}^{-{d}_{\alpha \beta }/\lambda })\cdot {e}^{-{d}_{\alpha \alpha }/\lambda }}=-\,\frac{{E}_{p}+\varphi }{{E}_{p}}{e}^{-(2{E}_{p}+\varphi )\varphi }$$

and is related to the chemical shift in AES measurements associated with compound layers with thickness *d*_*αβ*_. Based on Equation (7) with the constraint of the total coverage of Ni, we simulated the thicknesses of Ni-Ni layers and Ni-Si layers to match the chemical shift obtained in AES measurements as shown in Equation 7. The thickness of the NiSi layer can be assumed to represent the amount of Ni silicides. The simulated results for the amount of Ni silicides are shown in Fig. 3b as indicated by blue circles. The calculated amount of Ni silicides increases rapidly with increasing Ni coverage and reaches a saturated value around 1.8 ML. By assuming the existence of a layered structure of a Ni-Ni layer over a NiSi layer as illustrated in Fig. 3c, the simulated results for the amount of Ni silicides for a 1.8 ML thickness is in good agreement with the experimentally obtained thickness of the magnetic dead layer shown in Fig. 1e.

After summarizing the STM, AES and SMOKE results regarding the morphologies, chemical states and related magnetic properties, we can now revisit the schematic plots in Fig. 1e where three phases are resolved for different coverage regimes. For regime I (0 to 1.8 ML), a nonmagnetic behavior is observed, as indicated by the nonzero value on the coverage axis from the straight-line extrapolation of the SMOKE data, and is confirmed by both dark spots in the STM images and NiSi bond states by AES. In regime II (1.8 to 5 ML), no Kerr intensity is detectable (Fig. 1e) while the chemical state is transformed from Ni-Si to Ni-Ni as demonstrated by the AES findings in Fig. 3. The observation of nano-sized clusters with oval shapes by STM (Fig. 1b) is indicative of the formation of clusters with lowered Curie temperatures. In Regime III for a Ni coverage above 5 ML, ferromagnetic Ni films are produced. This is due to the increasing size of clusters, which are large enough to cross the superparamagnetic limit, thus forming a continuous film. The Curie temperature is well above room temperature.

**Figure 4:**
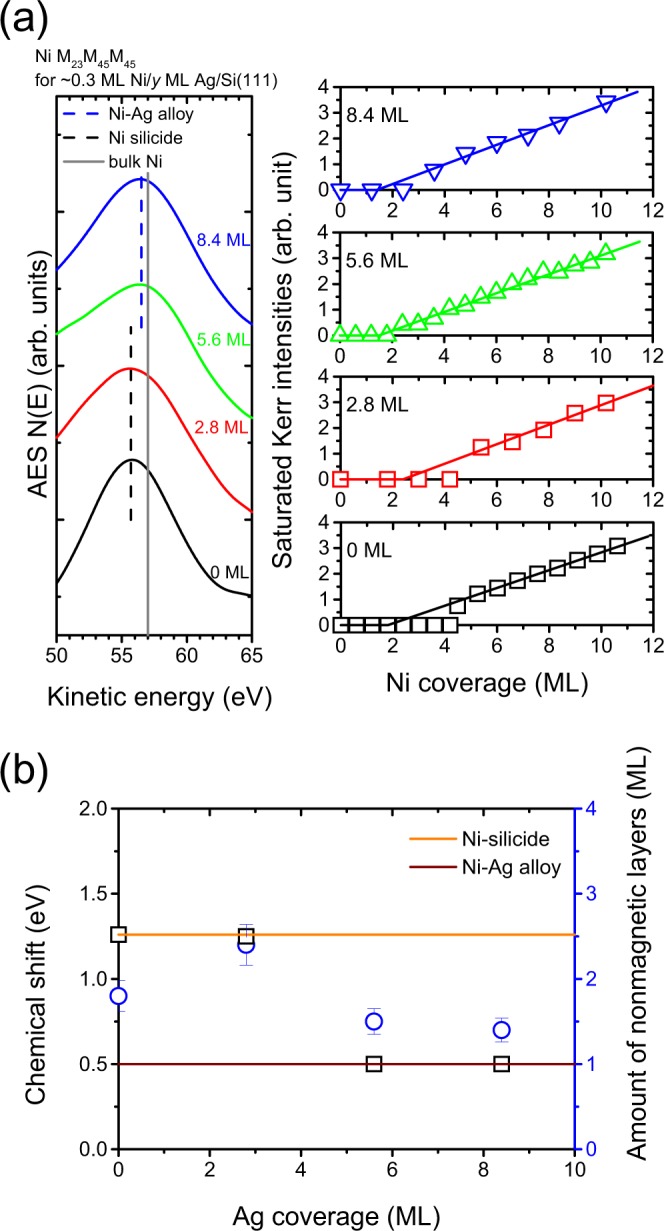
(**a**) Left panel: Auger spectra near the Ni M_23_M_45_M_45_ transition line for approximately 0.3 ML of Ni on Si(111) and Ag/Si(111) with an Ag layer thinner than 8.4 ML; right panel: saturated Kerr intensities versus Ni coverage for Ni/Ag/Si(111) with the Ag inserting layers being 0, 2.8, 5.6, and 8.4 ML. (**b**) The chemical shift (black squares) and the amount of nonmagnetic layers (blue circles) versus the Ag coverage.

Because of the high reactivity of a fresh Si surface, one possible route to passivate the Si surface so as to prevent chemical reactions between overlayers with the substrate is the insertion of an immiscible element. As an example of Co/Si(111), nonmagnetic silicide formation occurs at the interface while the insertion of Ag at Co/Si(111) functions to modify the interfacial conditions, thus preventing the formation of silicides at room temperature^15,17^. From the alloy phase diagram, each combination of Ag/Ni and Ag/Si is bulk immiscible^57^. We expected to see a similar behavior for the reduction of silicide for Ni/Si(111) by the insertion of Ag. However, with respect to Ag insertion, Co/Si(111) and Ni/Si(111) behaved differently. To explore this further, we examined the chemical states of Ni atoms at the initial stage of the deposition where the corresponding Auger spectra near the Ni M_23_M_45_M_45_ transition line are presented in the left panel of Fig. 4a. For 0.3 ML Ni/Si(111), the peak position of the Auger signals *N*(*E*) is located at 55.7 eV, which is related to Ni silicide states (black dash line), as discussed in Fig. 3. After the insertion of Ag, the Auger transition line shifts to a higher kinetic energy (blue dash line). The chemical shift of Ni M_23_M_45_M_45_ versus Ag coverage is summarized in Fig. 4b (black squares). For zero Ag coverage, the chemical shift for Ni M_23_M_45_M_45_ is 1.3 eV, indicative of the silicide state for Ni adatoms. For inserting Ag into layers thicker than 5.6 ML, the Ag layer is sufficiently thick that it blocks chemical interactions between Ni and Si at room temperature. Therefore, the chemical shift of 0.5 eV for Ni M_23_M_45_M_45_ corresponds to interactions of Ni and Ag atoms for approximately 0.3 ML of Ni at 5.6 and 8.4 ML Ag/Si(111). At the intermediate state for the 0.3 ML Ni at 2.8 ML Ag/Si(111), the chemical shift of Ni M_23_M_45_M_45_ at around 1.3 eV is close to that for Ni on pure Si(111). This suggests that the chemical state of Ni is closer to that for Ni silicides rather than Ni-Ag bonding.

The right panel of Fig. 4a shows the saturated Kerr intensities versus Ni coverage for Ni/Ag/Si(111) with a Ag layer thinner than 8.4 ML. As the Ni coverage increases, the Kerr intensities are initially zero followed by a linear increase for thicker Ni layers. The amounts of nonmagnetic layers are determined from the straight-line extrapolation of the data within the linear parts to a zero signal, and their values are summarized in Fig. 4b (blue circles). For inserting Ag in layers thicker than 5.6 ML, the amounts of the nonmagnetic layers are 2.0 ML. This is consistent with the quenched magnetic moments of the first layer due to electronic coupling effects as reported for Ni/Ag(111)^48^. For Ni/Si(111) without a Ag inserted layer, the thickness of the nonmagnetic layer is 1.8 ML. Because a 2.8 ML layer of Ag cannot completely cover the Si(111) substrate^17^, the nonmagnetic layer is predicted to be between 1.8 and 2.0 ML for Ni deposited on 2.8 ML Ag/Si(111) if we consider the areal contributions of the Ni silicide and the Ni/Ag interface. In the case of Ni on the Si(111) surface that is not completely covered by Ag, it was surprising to find that the amount of nonmagnetic layer is significantly enhanced to 2.4 ML, as shown in Fig. 4b. Because the position of the Auger transition line for a submonolayer of Ni on 2.8 ML Ag/Si(111) is close to that on pure Si(111), the nonmagnetic layer is related to the formation of Ni silicides. The enhancement in the amount of the nonmagnetic layer shows that the Ag atoms serve as a catalyst to accelerate the chemical interaction of Ni and Si atoms to form Ni silicides.

**Figure 5:**
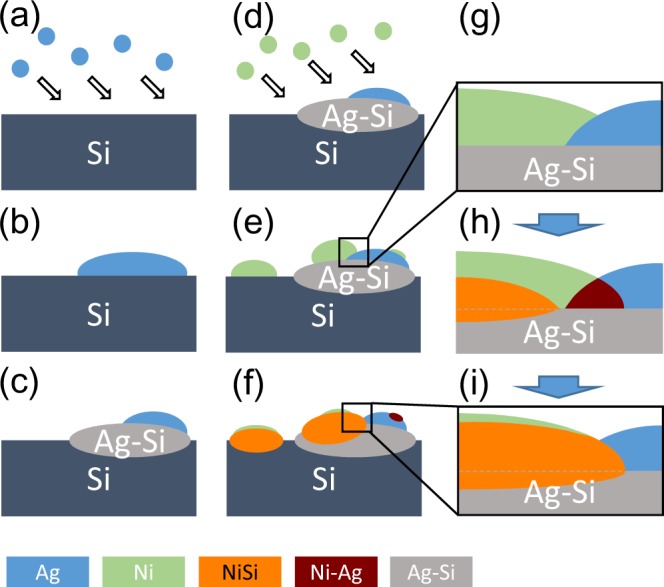
Schematic plots showing distribution of different species after Ni deposition with enhanced silicide formation.

From the experimental evidence showing a large chemical shift for submonolayer Ni on a few ML Ag/Si(111) (Fig. 4a), the formation of Ni silicides (Figs 2, 3 and 4a), and the enhanced dead layer (Fig. 4b), an interaction transferring mechanism for silicon atoms across the Ag layer is proposed to explain the enhanced silicide formation that occurs at room temperature. Schematic plots to elucidate the distribution of different species after Ni deposition showing an enhanced silicide formation are depicted in Fig. 5. From the discussions of formation enthalpies ΔH_Ag-Si_, ΔH_Ni-Ag_, and ΔH_Ni-Si_, the formation of Ag-Si particles plays a key role in enhancing silicide formation. Details of this are as follows. The rationale starts with the deposition of Ag on Si(111), as shown in Fig. 5a. A few MLs of Ag on the top of the Si(111) accumulate, with clusters being formed, as shown in Fig. 5b. The enthalpy of formation ΔH_Ag-Si_ is a negative value (−5.9 kJ/mol)^58^. From the thermodynamics point of view, the formation of Ag-Si bonding at the interface as well as the edges of the Ag islands is preferred after the deposition of Ag on Si(111) (Fig. 5c). The formation of Ag-Si particles plays a key role in enhancing silicide formation. For Ni deposited on the top of the submonolayer Ag/Si(111), Ni atoms can be adhered either on Si(111) patches or on the top of the Ag islands (Fig. 5d,e,g). Because of the high reactivity of the Si(111) surface, Ni silicide formation occurs on the Si(111) patches (Fig. 5f,h). On the top of the Ag islands, Ni-Si bonding is dominant as compared to Ni-Ag bonding because of the large negative enthalpies of formation for Ni silicides (${\rm{\Delta }}{H}_{NiS{i}_{2}}$ = −29.3 kJ/mol, ${\rm{\Delta }}{H}_{NiSi}$ = −38.7 kJ/mol)^52^. The interaction transfer occurs for Si atoms via Ag-Si bonding, un-bonded from Ag-Si, and finally bonding with Ni to form Ni silicides. This scenario causes the formation of Ni silicide on Ag clusters to be substantially enhanced (Fig. 5f,h,i) as evidenced by the large chemical shift shown in Fig. 4b.

## Conclusion

At the initial stage of Ni deposition on a Si(111) surface, dark defects with different sizes are observed and are related to the different chemical states of Ni silicides. This can be attributed to the strong bonding between Ni and Si atoms, NiSi is the relatively dominant species at the Ni/Si interface. Assuming that a Ni layer is formed over a NiSi layer with the total coverage as a constraint, we established a CSRC model that represents a practical method for determining the amount of ultrathin Ni silicides that are produced at the buried interface. Based on the morphologies, the chemical states and related magnetic properties of Ni/Si(111), the phases of nonmagnetic Ni silicides, Ni clusters with lowered Curie temperatures and ferromagnetic Ni films were resolved for different coverage regimes. For a submonolayer Ni deposited on a few monolayers of Ag/Si(111), the chemical state of Ni was found to be closer to that for Ni silicides rather than for Ni-Ag bonding. The finding that the amount of the nonmagnetic layer is enhanced shows that the Ag-Si particles serve as a catalyst that promotes the chemical interaction of Ni and Si with the formation of Ni silicides. An interaction transferring mechanism for silicon atoms across the Ag layer is proposed for the enhanced silicide formation. Based on the formation enthalpies Δ*H*_Ag-Si_, Δ*H*_Ni-Ag_, and Δ*H*_Ni-Si_, Ag-Si particle formation plays a key role in enhancing silicide formation via a specific interaction transfer mechanism at room temperature. The data and related mechanisms reported herein provide insights into strategies for producing ultrathin silicides at a buried interface.

## Methods

All experiments were performed under ultrahigh vacuum (UHV) conditions with a base pressure of around 1 × 10^−10^ torr. The Si(111) substrate was cleaned by repeated Ar^+^ ion bombardment and annealing treatments at 1250 K until a well-ordered 7 × 7 structure was obtained, as evidenced by STM and LEED. The LEED pattern and STM image for Si(111)-7 × 7 structure can be found in our previous report^15^. The purity of the surface was checked using AES. Ni atoms were evaporated from a resistively heated, high purity Ni coil (99.997%). Ag atoms were deposited on the surface from a well collimated evaporator using Ag rods with a high purity (99.999%) as the Ag source. The coverage of an overlayer was determined from the ratio of the intensities of the Auger signals of the overlayer and substrate atoms, and was double checked using a SYCON thickness monitor of a quartz balance. For overlayers on a Si(111) surface, one ML is equal to 0.08 nm as defined by the atomic density of substrate surface^14–16^. A He-Ne laser with a wavelength of 632.8 nm was used as the light source for the SMOKE measurements. The experimental components have been described in previous reports^13–17,22,23^.
